# Supplementary material for: Modelling nitrogen management in hybrid rice for coastal ecosystem of West Bengal, India
Source: PeerJ. 2023 Feb 15;11:e14903. doi: 10.7717/peerj.14903 (PMC9938656; doi:10.7717/peerj.14903)
Supplement: Supplemental Information 1 [file peerj-11-14903-s001.docx]

**Supplementary Table 1:** Bio-physical parameters modified in APSIM-Oryza crop module in simulating the hybrid rice cultivars under different nitrogen fertilizer doses.

| APSIM Code | Description | Unit | Default Value in APSIM-Oryza | Modified Value | Reference |
| --- | --- | --- | --- | --- | --- |
| RGRLMX | Maximum relative growth rate of leaf area | ℃d^-1^ | 0.0085 | 0.0125 | Bouman (2001) |
| RGRLMN | Minimum relative growth rate of leaf area | ℃d^-1^ | 0.0040 | 0.0080 | (Banerjee et al., 2022; Sarkar et al., 2022) |
| NMAXUP | Maximum daily N uptake | kg N ha^-1^ d^-1^ | 10 | 14 | (Banerjee et al., 2022) |
| FNTRT | Fraction N translocation from roots as (additional) fraction of total N translocation from stems and leaves (-) |  | 0.15 | 0.20 | Present Experimental value and  (Banerjee et al., 2022) |
| EFF | Table of light use efficiency (-; Y-value) as a function of temperature | ℃ | 0.54 0.54 0.36 | 0.54 0.60 0.46 | Present Experimental value |
| FSH | Table of fraction total dry matter partitioned to the shoot as a function of development stage (-; X value): | - | 0.50 0.75 1.00 1.00 | 0.47 0.80 1.00 1.00 | Present Experimental value |

Reference

Bouman, B. A. M. (2001). ORYZA2000: modeling lowland rice. IRRI.

Banerjee, H., Sarkar, S., Dutta, S. K., Garai, S., Ray, K., Zingore, S., Goswami, R., & Majumdar, K. (2022). Nitrogen management trade-offs in hybrid rice for agronomy, carbon, and energy efficiency. *Nutrient Cycling in Agroecosystems*, *0123456789*. https://doi.org/10.1007/s10705-022-10199-0

Sarkar, S., Gaydon, D. S., Brahmachari, K., Poulton, P. L., Chaki, A. K., Ray, K., Ghosh, A., Nanda, M. K., & Mainuddin, M. (2022). Testing APSIM in a complex saline coastal cropping environment. *Environmental Modelling & Software*, *147*(December 2020), 105239. https://doi.org/10.1016/j.envsoft.2021.105239


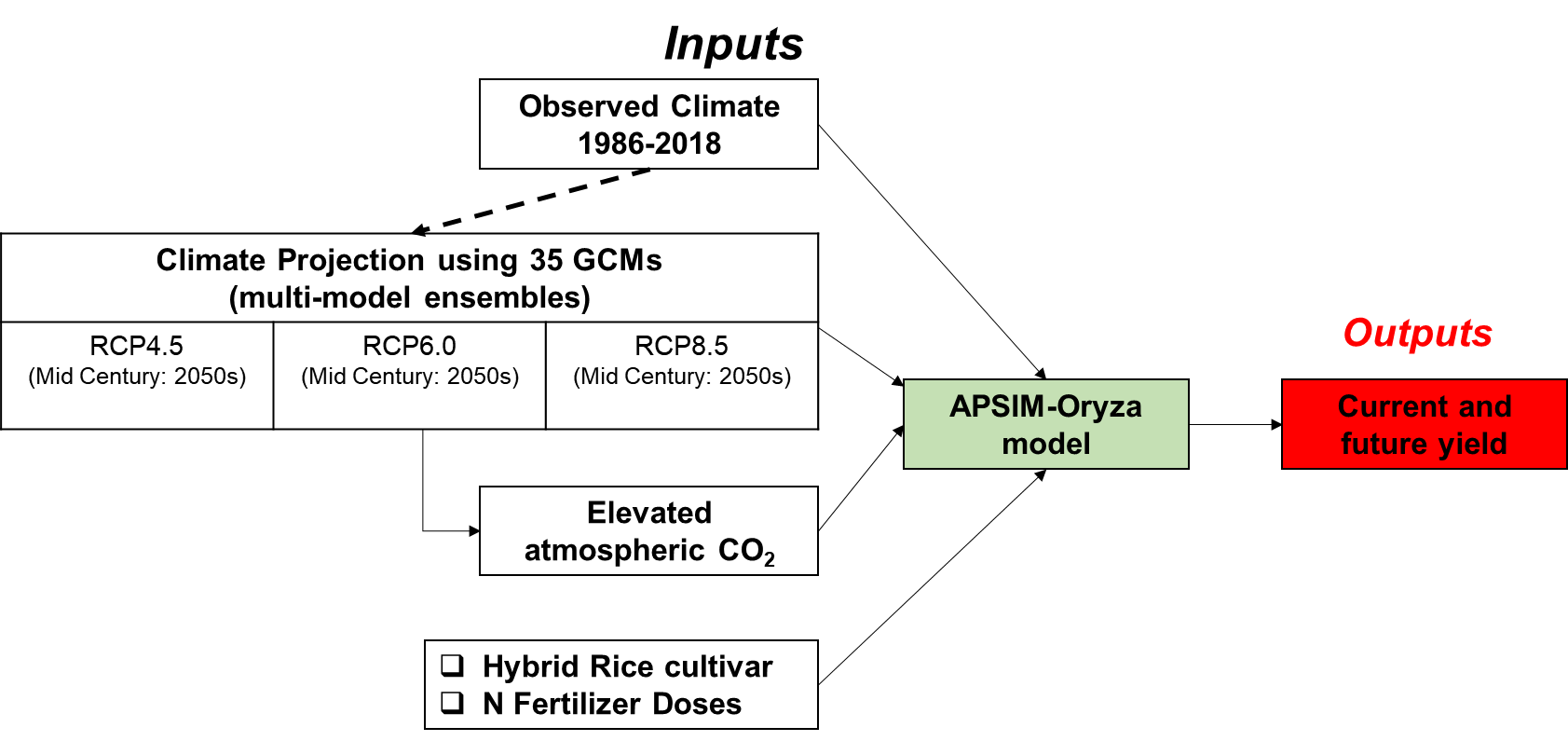


**Supplementary Fig. 1:** Schematic representation of climate change scenario

**Supplementary Fig. 2:** Effect of climate changes on long term simulated grain yield of of the hybrid rice grownat different nitrogen fertilizer doses: [Historical (1988-2018, RCP4.5, RCP6.0, and RCP8.5] (Each column represents the average grain yield of twenty years simulated grain yield, and vertical bars represent the standard deviation either side of the mean)
